# Report of a Case of Skin Grafting

**Published:** 1897-05

**Authors:** C. O. Weller

**Affiliations:** Austin, Texas


### TEXAS MEDICAL JOURNAL.

ESTABLISHED JULY, 1885.

PUBLISHED MONTHLY.—SUBSCRIPTION $2.00 A YEAR.

VOL. XII. AUSTIN, MAY, 1897. NO. 11.

### Original Contributions.

For the Texas Medical Journal.

### REPORT OF A CASE OF SKIN GRAFTING.

BY C. O. WELLER, M. D., AUSTIN, TEXAS.

Read before the Austin District Medical Society, March 8, 1897.

PLASTIC SURGERY, or that branch of surgical practice that consists in the restoration of lost tissues by the transplantation of integument, or other structures, from a more or less distant part of the body, has engaged the attention of surgeons from a very remote period. Prof. Gross says, "it is exceeding probable that one branch of plastic surgery has been practiced in India from time immemorial." In that country, the barbarous custom has existed for ages of punishing certain classes of criminals by cutting off their noses, and there can be no doubt that sympathy for these poor wretches gradually induced persons to turn their attention to the means of affording them relief. Hence arose rhinoplasty, or the operation of making new noses. According to Galen the ancient Egyptians were well acquainted with the art of rhinoplasty. I only make this brief reference to its history to show that the subject has been a matter for consideration for many centuries. I will say, without further historical allusion, that the methods of operating, have been improved from time to time, until now, under the present system of aseptic and antiseptic surgery, the operation is very successfully and satisfactorily performed. What I have to say on this occasion, will refer only to skin grafting; the operations of osteoplasty and "interpolating pieces of nerve trunks between the freshened sides of divided nerves," etc., having no bearing on the case to be reported. There are different methods of skin grafting with the nomenclature and technique of which you are all familiar, and it would not be of interest to you to mention them. I shall refer, only, to one method; that known as "Thiersh's method of skin grafting."

"The chief value of this method consists in the fact that it prevents the deformities that would result from the natural healing of a wound and the subsequent contraction of the scar." The operation is based upon his contention that the healing of a granulating surface results; first, "from the conversion of the soft, vascular granulation papillæ into dry cicatricial papillæ, by contraction of some of their elements, which have developed into young connective tissue cells. The result of this is to approximate the surrounding tissues, thus diminishing the area to be covered by epidermis; and secondly, by the covering of these papillæ with epidermic cells. Contraction has gone on as far as the laxity of the tissues will admit, and the capacity for developing new epidermic cells, by the margins of the wound being limited, the granulations of the unhealed central portion remain stationary, that is, vascular and soft. Few if any of their component cells having undergone development into connective tissue, the maximum of contraction has not yet been attained, but will be promptly reached if they are covered in with epidermis. Moreover any trivial mechanical or vascular irritation will give rise to exudation from the soft subjacent papillæ resulting in separation of the newly formed skin. Still further, microscopically, two layers of granulative tissue are discernible, the more superficial, possessing vertically disposed capillaries, the deeper containing a horizontal network of vessels, from which the former spring coursing through a structure more or less dense, according to its age, that is, of conversion into connective tissue.

A full removal of this upper, soft layer of granulations, yet capable of full contraction, must be effected to prevent cicatricial distortion and the risk of separation of the epidermis, these evils being avoided by laying the grafts directly upon the layer of granulations with horizontally disposed capillaries, to which layer the transplanted portions will become firmly adherent, and will remain so, undisturbed by cicatricial contraction." I have stated the principle underlying the Hirsch method, to refresh your memories on the subject, and will now make a brief report of the case to which application of his procedure was made.

Miss H., living in the country about two miles from Austin, was accidently shot in the face February 9th, 1895. The weapon was a shotgun loaded with small shot. The shot entered the right side of the face, just below the eye, and carried away the soft tissues, included in a line drawn horizontally from one-fourth of an inch below the margin of the lower eyelid to a point half an inch below the top of the ear, and another line drawn from the lower border of the right nostril across the cheek. The right molar bone was broken into numerous fragments. The whole bone, including its frontal, maxillary and zygomatic processes, was so comminuted that the removal of the entire structure, except its orbital process, was a necessity. The fragments of bone, enveloped as they were in the lacerated muscles, were carefully removed. All of the integumentary and muscular tissues that could be possibly saved were carefully stitched together. The nature of the wound through the lower eyelid caused some misgiving as to its probable result, fear being entertained lest it might result in cicatricial contraction in the healing process, and consequent eversion; fortunately, however, after stitching the lid to the integument below, there was union by "first intention," and the result was satisfactory beyond anticipation. As before mentioned, all of the integumentary and muscular tissues that could possibly be saved were stitched together; the wound was filled with moist iodoform gauze covered with absorbent cotton, securely retained by strips of adhesive plaster. The surroundings of the patient not being favorable for the treatment of a wound of this severity, nor for her personal comfort, her aunt kindly brought her to her home in town, where she could be better nursed and more convenient to her professional attendant. The wound was dressed twice a day for perhaps ten days or two weeks (I kept no notes, and write from memory) until all of the sloughs had separated or were removed with the knife or scissors. Boiled rain water and peroxide of hydrogen were used for cleansing the wound, which, after thorough washing, was packed with iodoform gauze and cotton, all held in place with adhesive plaster. There was fever during the first week, and some pain, each of which received the attention those conditions demanded. After the separation of the sloughs, granulations of a healthy nature sprang up, and the wound filled quite rapidly, so much so, that after the lapse of about five weeks preparatory treatment was instituted to put it in condition to be covered with grafts. This operation was considered necessary from what was evident the result would be without it; that is, a large disfiguring scar. The wound beginning to contract by the development of cicatricial tissue at its margins indicated the time had arrived for covering it with epidermic grafts. For a few days before the operation it was washed every other day with soapsuds and sublimate solution, and dressed with iodoform gauze saturated with balsam of Peru, and for one day before was covered with sublimate gauze. The thigh having been selected as the part from which the grafts would be taken, the day before was scrubbed with soapsuds, and with 1 to 1000 sublimate solution, and wrapped in sublimate gauze.

March 20th, 1895, thirty-nine days after the wound was received, was chosen as the day for operating. A large tin pail of salt solution was prepared, consisting of six parts of chloride of sodium to one thousand of water, boiled for one hour, and then kept warm by setting it in a larger vessel, and surrounding it with warm water. This salt solution, when needed, was emptied into four sterilized bowls, one of which was for the hands, one for the sponges, one for the gutta percha strips, and one to receive the grafts as they were cut. Strips of gutta percha tissue, three-quarters of an inch wide, and long enough to reach across the wound and lap on each side, were prepared. They were washed with soap seeds, and then with sublimate solution, and placed in salt solution, preparatory to being used. Sublimate gauze was wrung from the salt solution, preliminary to using for the inner dressing. The sponges were taken from carbolic solution and wrung out in the salt solution. The patient was now placed on the table, chloroform administered, the wound uncovered, the granulations carefully but effectually scraped away, and cicatrized borders removed with the knife. The raw surface was then washed with the salt solution, and a compress of gauze at once applied, held firmly in place by a bandage. This was done to stop oozing of blood from the raw surface. The bichloride towels were thrown back from the thigh, the sublimate bandages removed, and my assistant grasped the skin of the thigh with both hands and drew it tense transversely. The operator placed the thumb of the left hand on the tense skin at the upper part of the thigh, in order to make a little longitudinal tension, and with the right hand cut the grafts.

As soon as they were cut they were dropped into a bowl containing the warm salt solution. Oozing from the wound having ceased, the grafts were applied so that they overlapped each other to a small extent, also, the surrounding skin. "Overlapping does no harm, for the superficial edge of a graft will die from lack of nutrition, and come away leaving a mere line of union visible." The whole raw surface now being covered with epidermis, was gently douched with the salt solution, and the strips of gutta percha tissue applied. Upon these was placed damp salt gauze, and over this a sheet of gutta percha tissue, to maintain the moisture, then sublimate gauze and cotton, all held in place by strips of adhesive plaster. The dressing was changed every two days for the first eight days. At the end of six days the gauze was allowed to dry by omitting from the dressing the sheet of gutta percha tissue. This was done to prevent too great maceration of the cuticular layer of the grafts. After the lapse of ten days the strips of gutta percha tissue were removed and the grafts were found adherent in all (except two small places where they were too thick), and presenting a healthy appearance. The parts were then covered with a light dressing for two or three weeks, when all treatment ceased. This little operation was performed, as nearly as could be, with absolutely aseptic precautions. It was done according to instruction, for its performance, as given in the appendix to the "Reference Hand Book of the Medical Sciences."

When operating on a part where it is not desirable to have hair growing—as in the present instance, the face of a woman—great care must be exercised in cutting the grafts, lest we cut too deep and bring away the hair bulbs. If this is done, we will have hair growing from an undesirable locality. Moreover, a thick graft is not as likely to give as good a result as the very thin ones. It should include nothing below the papillary layer. In the case reported the patient came partially from under the influence of the anæsthetic, and moved her thigh slightly, thereby causing the razor to cut a little too deeply in one or two places. The result is, there are two small places, where those thick grafts were put, where there is a little cicatricial tissue, and a few hairs grow from the transplanted tissues. Yet, altogether, the result is quite satisfactory, and in attestation of this statement, I now present the lady who was operated on, and you can see for yourselves, whether or not the operation is a success.

